# Titanium Dioxide Promotes the Growth and Aggregation of Calcium Phosphate and Monosodium Urate Mixed Crystals

**DOI:** 10.3390/cryst14010011

**Published:** 2023-12-22

**Authors:** Onyebuchi C. Ukaeje, Bidhan C. Bandyopadhyay

**Affiliations:** Calcium Signaling Laboratory, Research Service, Veterans Affairs Medical Center, 50 Irving Street, NW, Washington, DC 20422, USA

**Keywords:** titanium dioxide, calcium phosphate, monosodium urate, crystal aggregation, dissolution kinetic models, gout disease

## Abstract

The increased utilization of titanium dioxide (TiO_2_) nanoparticles (TNPs) in various industrial and consumer products has raised concerns regarding its harmful effect due to its accumulation within the different systems of the human body. Here, we focused on the influence of TNPs on the growth and aggregation of two crucial crystalline substances, calcium phosphate (CaP) and monosodium urate (MSU), particularly its implications in gout disease. In this study, we adopted microscopic techniques and generated kinetic models to examine the interactions between TNPs, CaP and MSU, and crystallization, under controlled laboratory conditions. Our findings reveal that TNPs not only facilitate the growth of these crystals but also promote their co-aggregations. Crystal dissolution kinetics also exhibit that an increase in TNPs concentration corresponds to a reduction in the dissolution rate of CaP and MSU crystals in presence of the dissoluting agent hydroxycitrate (Hcit). These observations suggest that TNPs can stabilize CaP+MSU mixed crystals, which underscores the significance of TNPs’ exposure in the pathogenesis of gout disease.

## 1. Introduction

Titanium dioxide (TiO_2_) is widely utilized in diverse applications, including cosmetics [1–4] and as a food additive (E171) due to its white pigment properties [5–8], enhancing product aesthetics and texture. This growing usage of materials containing TiO_2_ nanoparticles (TNPs) causes unavoidable human exposure, which leads to the possibility of adverse effects of TiO_2_.

TNPs can penetrate the human body via several different pathways, including oral, dermal, and inhalation routes [9,10]. The accumulation of TNPs in the blood is a growing concern in the field of nanotoxicology [9,11]. Recent studies have shown that exposure to TNPs, commonly used in various consumer products, can lead to their gradual uptake into the bloodstream [12]. This phenomenon is linked to the smaller size and higher surface areas of these nanoparticles, enabling their penetration through the skin or respiratory system and subsequent distribution in the bodily systems through the blood stream [13,14].

Gout, a debilitating form of inflammatory arthritis, is closely associated with the deposition of monosodium urate (MSU) and calcium phosphate (CaP) crystals in affected joints and tissues, contributing to the complex pathogenesis of this disease [15–17]. The prevalence of gout varies from <1% to 6.8% with an incidence of 0.58–2.89 per 1,000 people per year [18]. MSU crystals are considered the primary culprits in initiating acute gout flares, since the interaction of these needle-like crystals initiates immune cells to trigger a robust inflammatory response characterized by elevated levels of interleukin-1β (IL-1β) [19]. However, recent research has highlighted the role of CaP crystals as a form of calcium pyrophosphate dihydrate (CPPD), which is implicated in the development of the symptoms of gout [20,21]. Such condition of CPPD deposition usually affects patients over 65 years old, with thirty to fifty percent being present in patients over 85 years old [21]. A cross-sectional study has also shown that ~2000 cases of CPPD in US veterans reported a point prevalence of 5.2 per 1000, with an average age of 68 years in which 95% are male [22]. The coexistence of MSU and CPPD crystals in gout patients has been reported, complicating the clinical presentation and management of the disease [23]. While the diagnosis of calcium pyrophosphate with MSU crystals is rare, it was found in 7.7% samples where patients had knee joints showing intermediate to high grade osteoporosis and may have higher detections with improved diagnostic methods [23]. We propose that the presence of TNPs can exacerbate the inflammatory response, which may add an additional layer of complexity towards the pathophysiology of gout.

The simultaneous co-crystallization of TNPs, MSU and CaP within the joints and tissues can significantly exacerbate the severity of the condition. CaP is not only a family of natural minerals, but also includes biominerals in humans, which are the main inorganic component of hardy tissues such as bone and teeth [24]. It is possible that the pH of the local microenvironment of bone can be affected by the released calcium (Ca^2+^) and phosphate (PO_4_^3−^) ions, which then may influence the viability of osteoblasts and osteoclasts [24]. The supersaturation of Ca^2+^ and PO_4_^3−^ can act as a nucleation site for the initiation of biomineralization [25], which may be favorable for other crystals to aggregate or co-crystallize at the site. We propose that TNPs can influence the stabilization of these crystals, favoring the aggregation and growth of mixed crystals.

While the TNPs have not been shown to be involved in the pathogenesis of gout disease, it is possible that the trace amount of TNPs may be available in the tissue microenvironment due to their widespread use. Moreover, TNPs have the potential to act as foreign bodies, and hence the interaction of TNPs with both MSU and CaP crystals can intensify the inflammatory response and cause persistent pain and joint damage [26,27]. The presence of these multiple crystal types may complicate the clinical presentation, diagnosis, and management of gout. Hence, a comprehensive understanding of the interplay between TNPs, MSU and CaP crystals could have the potential to improve diagnosis and management in patients with mixed-crystal depositions. Here, we aimed to explore the potential role of TNPs in the promotion of CaP and MSU crystal growth and aggregation while inhibiting their dissolutions.

## 2. Materials and Methods

### 2.1. Materials

TNP powder (<100 nm), sodium phosphate dibasic (Na_2_HPO_4_), sodium phosphate monobasic (NaH_2_PO_4_), calcium chloride (CaCl_2_), methylene blue dye (95%), hydroxyl citrate (Hcit), alizarin red (AR), and uric acid sodium salt were acquired from Sigma-Aldrich (St. Louis, MO, USA). Without additional purification, all reagents were used as purchased.

### 2.2. CaP, MSU and Mixed-Crystal Synthesis

CaP and MSU crystals were synthesized as described in our previous study with a few modifications [28]. Briefly, saturated CaP solution was prepared by mixing solutions of 0.9 mM Na_2_HPO_4_, 2.4 mM CaCl_2_, and 5.8 mM NaH_2_PO_4_. Mixed crystals were prepared by adding solutions of 2.4 mM CaCl_2,_ 0.9 mM Na_2_HPO_4_, 5.8 mM NaH_2_PO_4_, and 12 mM MSU solution and varying concentrations of TiO_2_ (0.5 mM. 1 mM and 3 mM). Monosodium urate crystals were prepared by first dissolving uric acid sodium salt in 1x HBSS and then centrifuged to collect the precipitates [28]. All saturated solutions and crystals were prepared in 1x Hank’s Buffered Salt Solution (HBSS) and at room temperature (RT; 25 °C). For mixed crystals, the solutions were stirred for 30 min at RT, followed by a 5 min, 10,000 rpm centrifugation. The supernatant was then discarded, and the crystals were washed twice with 1x HBSS. Other than the dissolution in di-H_2_O and Hcit, the pH of all tests involving crystal formation were kept at 4.3 using HBSS.

### 2.3. Alizarin Red Staining

As described, AR staining was carried out to look for the presence of CaP crystals [28]. Briefly, equal amounts of AR dye (pH 4.3) were added to sample solutions containing preformed CaP crystals, and the samples were incubated for 30 min at 37 °C for examination under high resolution light microscopy. Using ImageJ (version 1.37; National Institutes of Health, Bethesda, MD, USA), stained pictures were measured in line with our previously published study [29]. Stained crystal sizes were measured and quantified as “crystal index” using ImageJ as described [29,30]. The freehand tool was used to create irregularly shaped selections in ImageJ to trace the crystals. Particle sizes of less than 10 μm were excluded from the overall crystal size average [29].

### 2.4. Methylene Blue Staining

**Figure 1.**
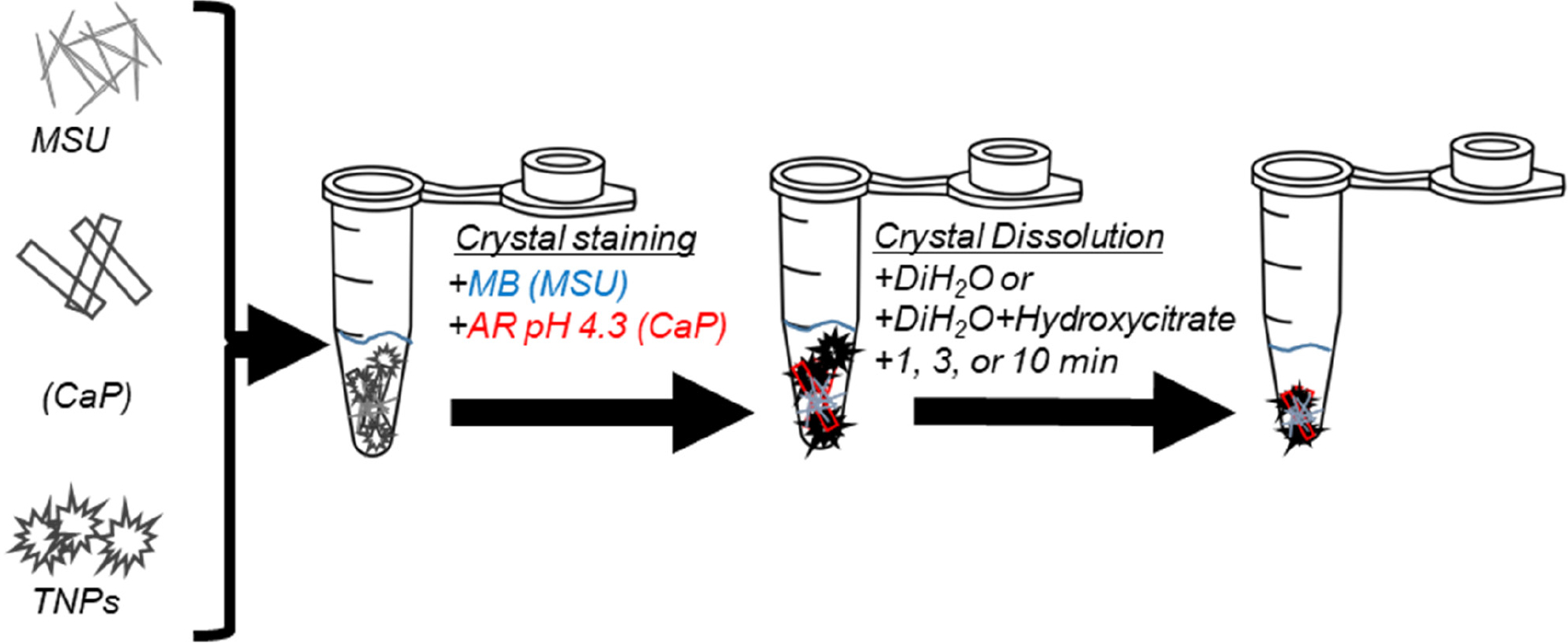
Schematic diagram for crystal staining of monosodium urate (MSU; methylene blue) and calcium phosphate (CaP; AR pH 4.3) in the presence of TNPs, and dissolution of MSU, CaP and/or TNPs in diH_2_O or Hcit in 1, 3 and 10 min (mins) time-lapse intervals.

The method adopted for methylene blue staining for the identification of MSU was adopted as described [28]. Briefly, equal amounts of methylene blue dye were added to the mixed-crystals solutions containing preformed MSU crystals; followed by incubation for 30 min at 37 °C. Stained MSU crystals were identified with the light microscopy and images acquired were measure with the ImageJ software in line with our previous study [29]. A schematic representation of the methods adopted for the differential staining of the crystals is shown in Figure 1.

### 2.5. Time-Dependence Crystal Formation and Dissolution

Saturated solutions were incubated for 0, 1, 3 and 10 min to examine changes in crystal development at RT. Preformed crystals were incubated at RT with di-H_2_O or Hcit for 0, 1, 3 and 10 min to assess crystal retention by counting the number of crystals still in solution [28]. Crystals were quantitatively measured after being differently dyed with AR pH 4.3 for CaP and methylene blue for MSU for each experiment.

### 2.6. Quantification of Crystal Index

Stained crystal sizes were measured and quantified as crystal indexes using ImageJ (NIH, USA) software in line with study by Ezell et al. [29]. The freehand tool was used to create irregularly shaped selections in ImageJ to trace the crystals. Types of crystals were identified based upon the staining and categorized accordingly: AR pH 4.3 stained for CaP, and methylene blue stained for MSU crystals. Particle sizes of less than 10 μm were excluded from the overall crystal size average. The crystal quantifications were repeated several times (*n* > 3).

### 2.7. Statistical Analysis

Statistical analyses were performed using the Origin 6.1 software. Data were expressed as mean ± SD. Comparisons were assessed using student’s *t* test. Multiple group comparisons were performed using one-way ANOVA, followed by the post hoc Tukey test.

## 3. Results and Discussions

### 3.1. TNPs Facilitates CaP and MSU Crystal Coaggregation

**Figure 2.**
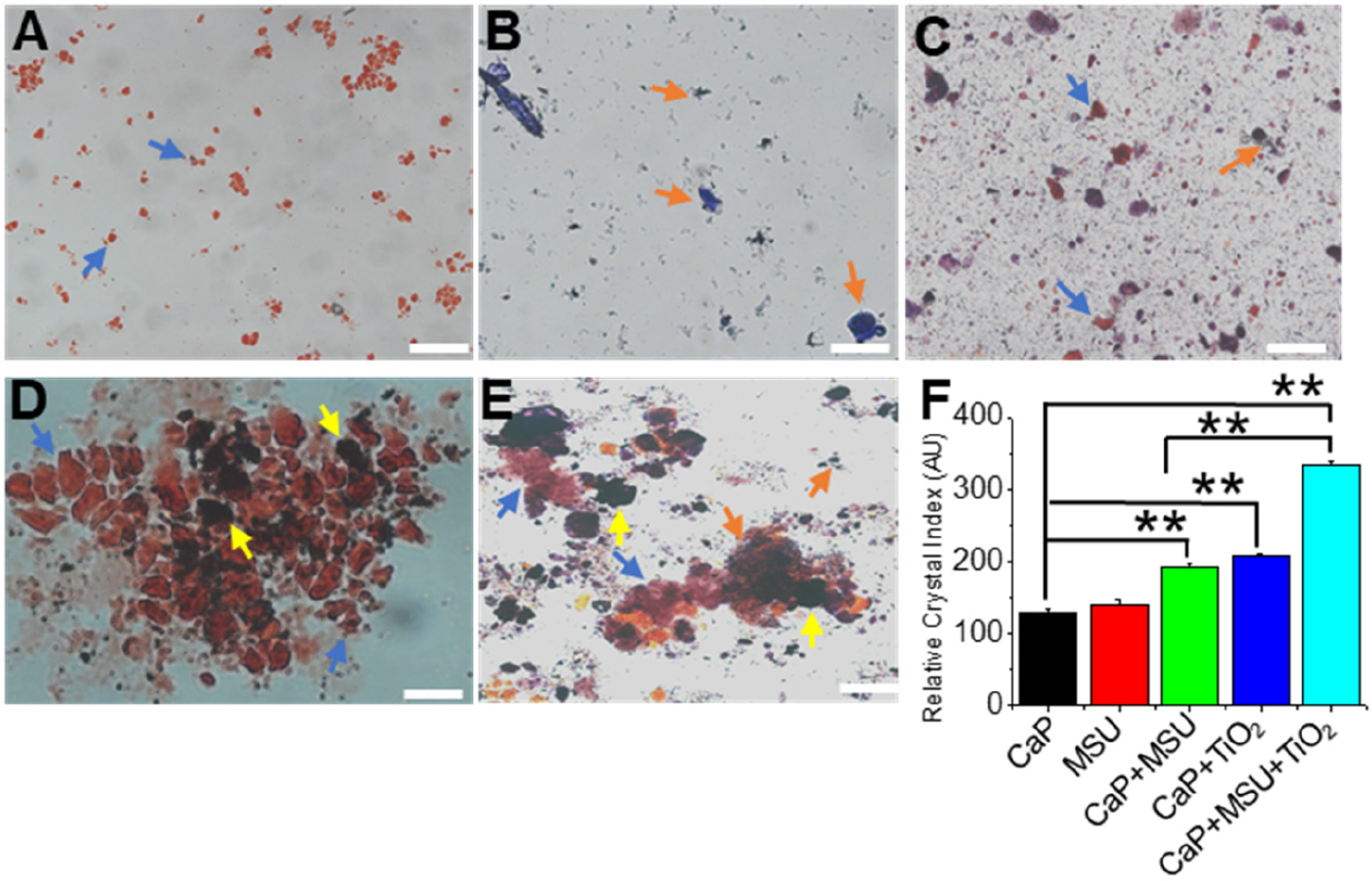
Differential staining and microscopic analysis of calcium phosphate (CaP), monosodium urate (MSU) and titanium dioxide (TiO_2_) nanoparticles’ (TNPs) interactions. (**A**) Individual CaP crystals, (**B**) individual MSU crystals, (**C**) CaP+MSU co-crystallization and (**D**) CaP+TNPs mixed-crystal sample showing minimal staining of TNPs by AR pH 4.3, and (**E**) mixed-crystal sample displaying enhanced aggregation in the presence of TNPs. Differential staining was conducted using AR pH 4.3, with methylene blue counterstaining. The results indicate a substantial increase in crystal aggregation with TNPs, particularly evident in the CaP+MSU+TNPs crystallization, suggesting a pivotal role for TNPs in promoting interactions and aggregation among CaP and MSU crystals. (**F**) Relative crystal sizes were quantified using ImageJ and were represented as a bar diagram for mean + S.E.M. of *n* ≥ three independent experiments (** *p* < 0.01). Scale bar (white) = 50 μm. Blue (CaP), orange (MSU) and yellow (TNPs) arrows are depicted in the images. (For interpretation of the references to color in this figure legend, the reader is referred to the web version of this article).

In this study, we presented the significant increase in the coaggregation of mixed crystals by TNPs as evidently presented in Figure 2. The experimental approach taken in this study was multifaceted and aimed at elucidating the interactions and crystal formation dynamics of CaP, MSU, and TNPs. To begin, we conducted a differential staining procedure using AR pH 4.3, with methylene blue utilized as a counterstain. Remarkably, TNPs exhibited minimal staining and appeared dark within the mixed-crystal samples, as shown in Figure 2D. The stark contrast in staining patterns between TNPs and CaP or MSU crystals underscored the distinctive role of TNPs in this system.

Furthermore, our microscopic observations, as presented in Figure 2A–E and corroborated by AR pH 4.3 and methylene blue staining, revealed a compelling growth-promoting phenomenon. We noted a substantial increase in the aggregation and growth of CaP and MSU crystals in the presence of TNPs, indicative of a significant interplay between these crystal types. The synergistic effect of TNPs on crystal aggregation was particularly pronounced in the case of CaP+MSU+TNPs crystallization, as illustrated in Figure 1E. These findings provide convincing evidence that TNPs plays a pivotal role in facilitating interactions between CaP and MSU crystals, ultimately leading to enhanced aggregation within the mixed-crystal formations.

### 3.2. TNPs Led to the Prolonged Retention of Mixed Crystals over Time in the Presence of di-H_2_O or HCit (Crystal Inhibitor)

**Figure 3.**
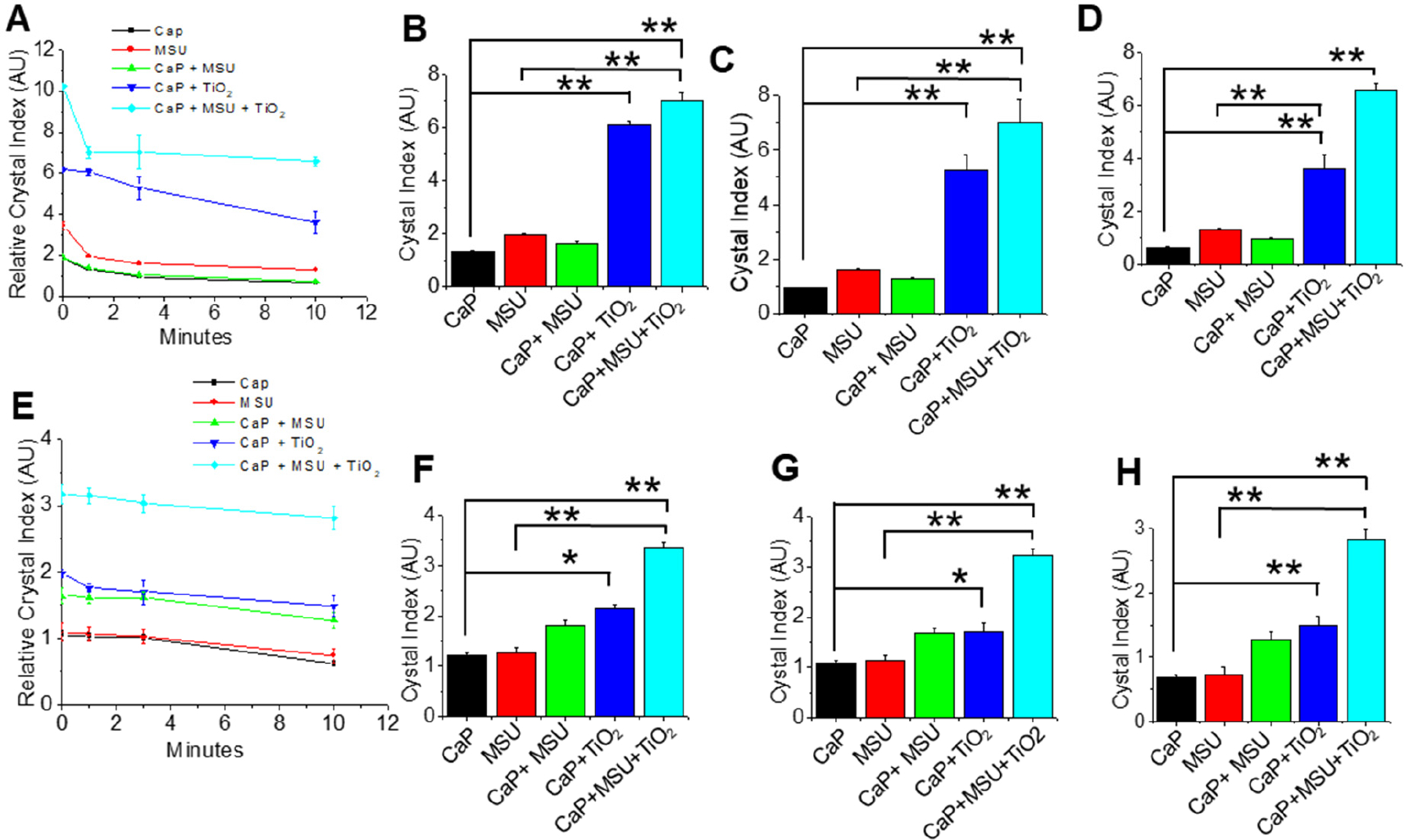
(**A**) Time-dependent dissolutions of calcium phosphate (CaP), monosodium urate (MSU), and titanium dioxide (TiO_2_) nanoparticles’ (TNPs)-mixed crystals in di-H_2_O. Time-dependent dissolution in di-H_2_O at (**B**) 1 min, (**C**) 3 min and (**D**) 10 min timepoints. (**E**) Time-dependent dissolutions of CaP+MSU and TNPs (CaP+MSU) in Hcit at (**F**) 1 min in Hct, (**G**) 3 min in Hcit and (**H**) 10 min in Hcit. Relative crystal sizes were quantified using ImageJ and were represented as a bar diagram for mean + S.E.M. of *n* ≥ three independent experiments (* *p* < 0.05 and ** *p* < 0.01).

Our initial observations show that TNPs had the potential to increase the formation of mixed crystals composed of CaP and MSU. This prompted us to investigate whether TNPs could also stabilize these mixed CaP crystals. To assess this, we conducted several dissolution experiments on CaP, MSU, CaP+MSU, CaP+TNPs, and CaP+MSU+TNPs crystals in an aqueous environment (di-H_2_O) by monitoring their sizes at various time points (0, 1, 3 and 10 min) (Figure 3A–D). We observed that CaP crystals alone displayed the highest susceptibility to dissolution, particularly within the first minute, and this trend continued over the 10 min duration (Figure 2A). Furthermore, the CaP+MSU crystals exhibited a significantly higher dissolution rate compared to the CaP+TNPs group over the entire 10 min period (Figure 2A,D). Notably, the CaP+MSU+TNPs mixed crystals maintained significantly larger sizes than both CaP+MSU and individual crystals until the 10 min mark (Figure 3A–D). Interestingly, while the inclusion of TNPs reduced dissolution, the rate of dissolution of mixed MSU+CaP+TNPs crystals was slightly higher than that of CaP+TNPs mixed crystals (Figure 3B–D), a trend that reversed in the absence of TNPs, as depicted in Figure 3.

In the presence of HCit, all crystal groups started to dissolve, even when TNPs were present (Figure 3E–H). As expected, the CaP+MSU+TNPs mixed crystals displayed a slower dissolution rate when HCit was introduced. However, under the influence of HCit, individual crystals and CaP+MSU mixed crystals dissolved more rapidly than TNP-mixed crystals (Figure 3E–H). It is worth noting that the dissolution rates did not significantly differ (*p* > 0.05) among the CaP, MSU, and CaP+MSU groups throughout the 10 min duration (Figure 2E). These results underscore the fact that TNPs play a role in stabilizing mixed-crystal aggregates, even in the presence of HCit. This novel finding may offer a valuable insight into the potential implication on the impact of TNPs on gout disease progression.

### 3.3. TNPs Inhibited Mixed-Crystal Dissolution in a Concentration-Dependent Form

**Figure 4.**
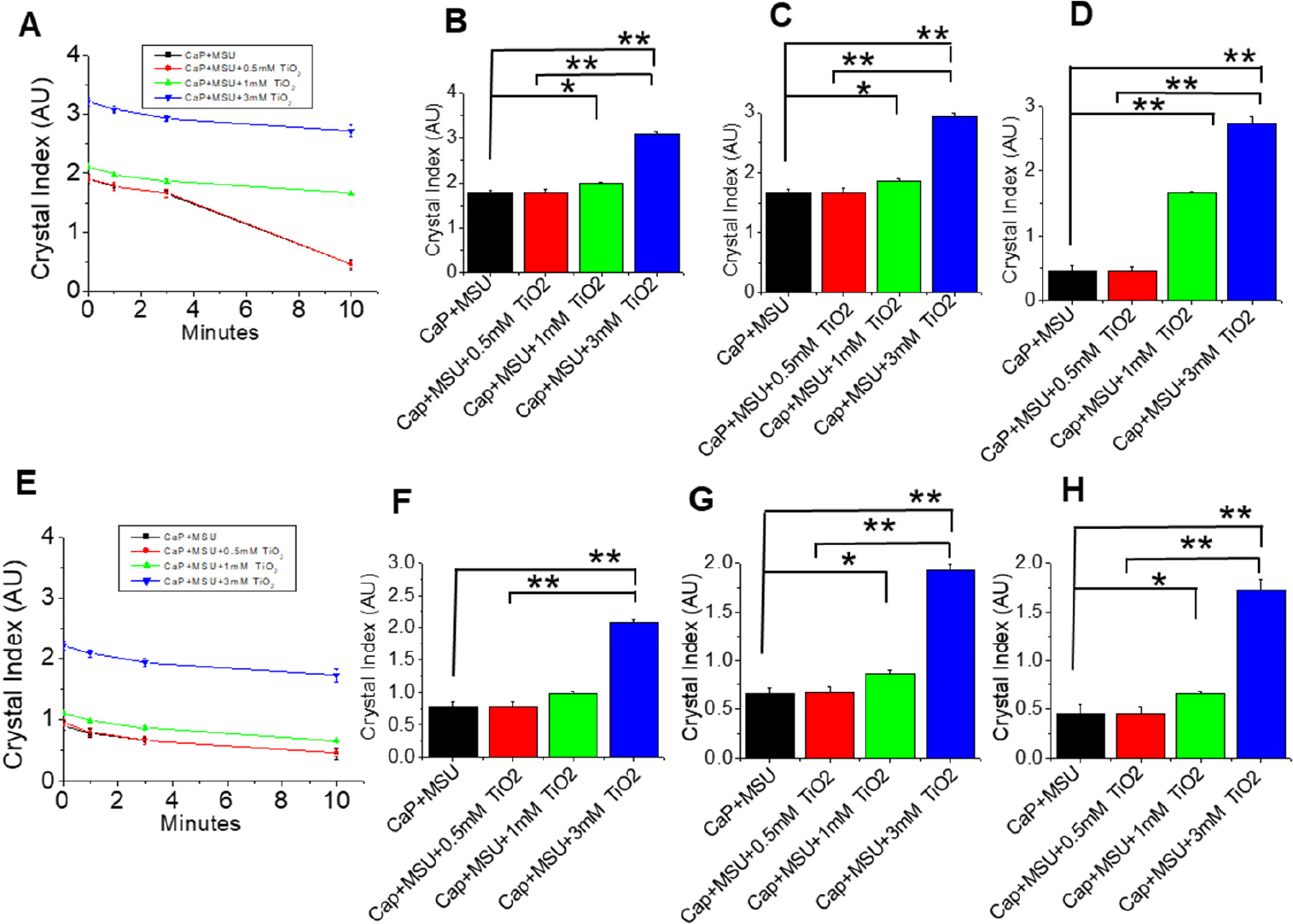
Concentrations and time-dependent dissolution of mixed-crystals involving calcium phosphate (CaP), monosodium urate (MSU) and different concentrations of titanium dioxide (TiO_2_) nanoparticles (TNPs) in di-H_2_O. (**A**) Dissolution experiments at various TNP concentrations (0 mM, 0.5 mM, 1 mM, and 3 mM) over (**B**) 1 min, (**C**) 3 min and (**D**) 10 min exposure periods. (**E**) Time-dependent dissolutions of mixed-crystals with different TNPs concentrations and the addition of HCit over (**F**) 1 min, (**G**) 3 min and (**H**) 10 min exposure times. The results illustrate the concentration-dependent effects of TNPs on mixed-crystal dissolution and the impact of HCit. Relative crystal sizes were quantified using ImageJ and were represented as a bar diagram for mean + S.E.M. of *n* ≥ three independent experiments (* *p* < 0.05 and ** *p* < 0.01).

In this section, our goal was to further investigate whether the low dissolution of mixed crystal in the presence of TNPs is a function of TNP concentration. Hence, we performed CaP+MSU+TNPs crystal dissolution experiments involving different concentrations of TNPs (0 mM, 0.5 mM, 1 mM, and 3 mM) in the presence of di-H_2_O (Figure 4A–E). The quantification of the crystal sizes showed significant differences (*p* < 0.05) in crystals between CaP+MSU and mixed crystals in the presence of 1mM TNPs for 10 min exposure time (Figure 3A–D). A profound significant difference (*p* < 0.01) was observed at 10 min exposure between CaP+MSU and mixed crystals + 1 mM TNPs. A notable increase in the concentration of TNPs up to 3 mM significantly (*p* < 0.01) reduced the rate of crystal dissolution compared to the rest of the mixed-crystals (Figure 4A–D). This signifies that high concentrations of TNPs increase crystal growth and aggregation and consequently results in low crystal dissolution over time. Similarly, we investigated the TNPs’ concentration dependence in relation to crystal dissolution in the presence of Hcit. There was no significant difference between CaP+MSU and CaP+MSU+TNPs at TNP concentrations of 0.5 mM and 1 mM at 1 min exposure time (Figure 3E). However, there was significantly low crystal dissolution at 3 mM TNPs (*p* < 0.05) compared to the rest of the mixed-crystals at 1 min exposure time (Figure 4E). At 3 min and 10 min exposure, significant reduction in dissolution were observed at TNP concentrations of >1 mM (Figure 4E,G,H).

### 3.4. Crystal Dissolution Kinetics

The rate at which a solid dissolves, as per the Noyes–Whitney equation [31], relies on various factors, including the solubility of the substance, the concentration of the solute in the solution at a given moment, the diffusivity in the solution volume, and the surface area of the solid material [31]. When dealing with CaP and MSU crystals, the sizes of their particles and the binding of secondary amorphous particles such as TiO_2_ can significantly influence the dissolution rate.

**Figure 5.**
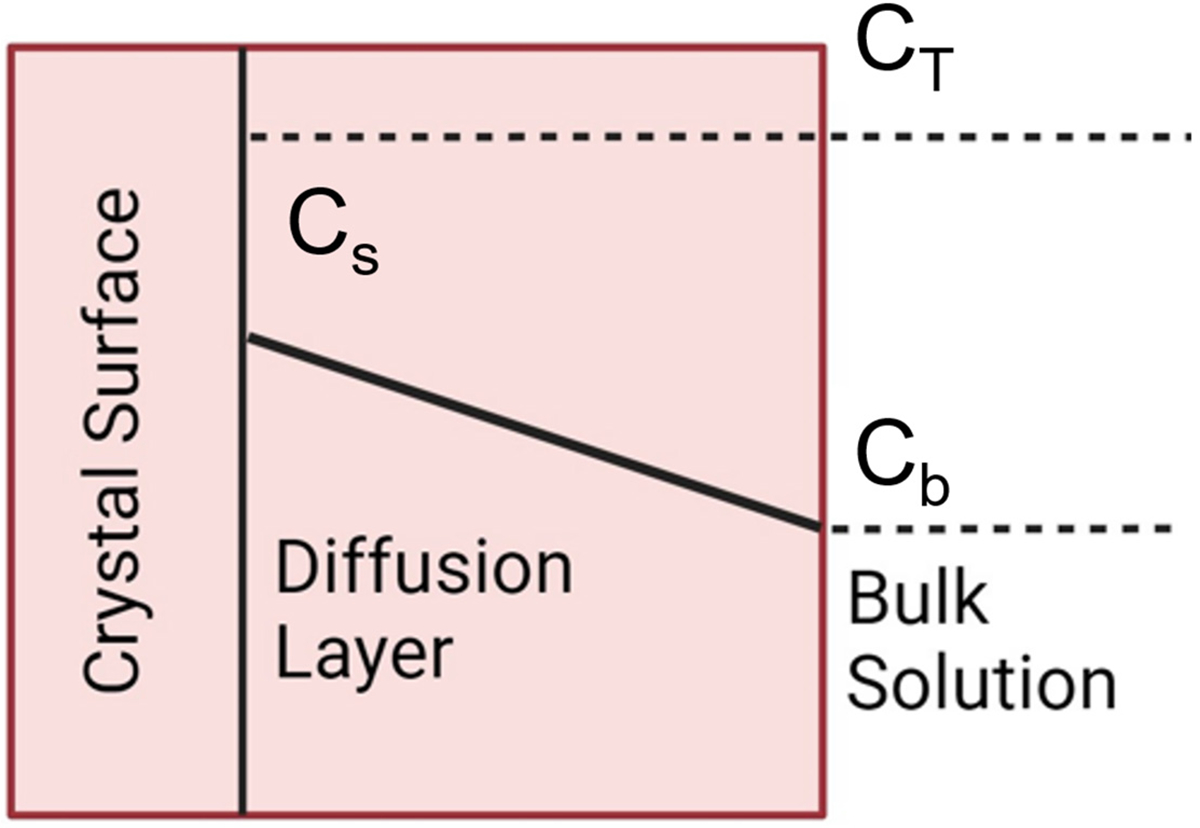
Dissolution kinetics model.

Dissolution, essentially the reverse process of crystal growth, involves two primary steps: (1) the reaction at the surface and the disintegration of surface species, and (2) the transfer of these species into the bulk solution across the diffusion layer, as illustrated in Figure 5. Assuming that the surface reaction rate $\left(S_{r}\right)$ and the mass transfer rate $\left(m_{r}\right)$ are directly proportional to the surface area of undissolved crystals per unit volume of the solution (S), and utilizing the concentration difference as a driving force, two equations can be derived as follows:

(1) $$S_{r}=k_{r}r\left(C_{T}-C_{S}\right)$$

(2) $$m_{r}=k_{m}r\left(C_{S}-C_{b}\right)$$

where $C_{T}$ represents the solubility of the solute at a constant temperature (25 °C), $C_{S}$ denotes the solute concentration at the crystals’ surface, and $C_{b}$ signifies the concentration of the solution in the bulk. Additionally, $k_{r}$ and $k_{m}$ correspond to the rate constants for surface reactions and the rate of mass transfer, respectively.

With consideration to the concentration of TNPs (V), one can also presume that in conditions where the system is nearly constant over time, the constant rate of dissolution $\left(K_{r}\right)$ is equivalent to both the rate of the surface reaction $\left(S_{r}\right)$ and the rate of mass transfer $\left(m_{r}\right)$.

(3) $$k_{r}=-\frac{dm}{V\;dt}=m_{r}=S_{r}$$

When considering the undissolved crystal’s mass (m), along with the concentration of TNPs (V) and the time it takes for dissolution (t).

A combination of Equations (1)–(3) gives.

(4) $$k_{r}=-\frac{dm}{V\;dt}=kS\left(C_{T}-C_{S}\right)$$

(5) $$k=\frac{1}{1/k_{m}\;+\;1/k_{s}}$$

The mass of the remaining crystals can be determined through a mass balance as follows, provided that $C_{b}=0$ at the initial time, t=0.

(6) $$m=m_{o}-C_{b}V$$

where $m_{0}$ is the initial mass of crystals. Combining Equations (4) and (5),

(7) $$k_{r}=-\frac{{dC}_{b}}{dt}=kS\left(C_{T}-C_{S}\right)$$

To depict the dissolution process in a diffusion-controlled scenario, one can employ a Noyes−Whitney-type expression.

(8) $$d_{r}=-\frac{dm}{V\;dt}=\frac{DS}{h}\left(C_{T}-C_{S}\right)$$

**Figure 6.**
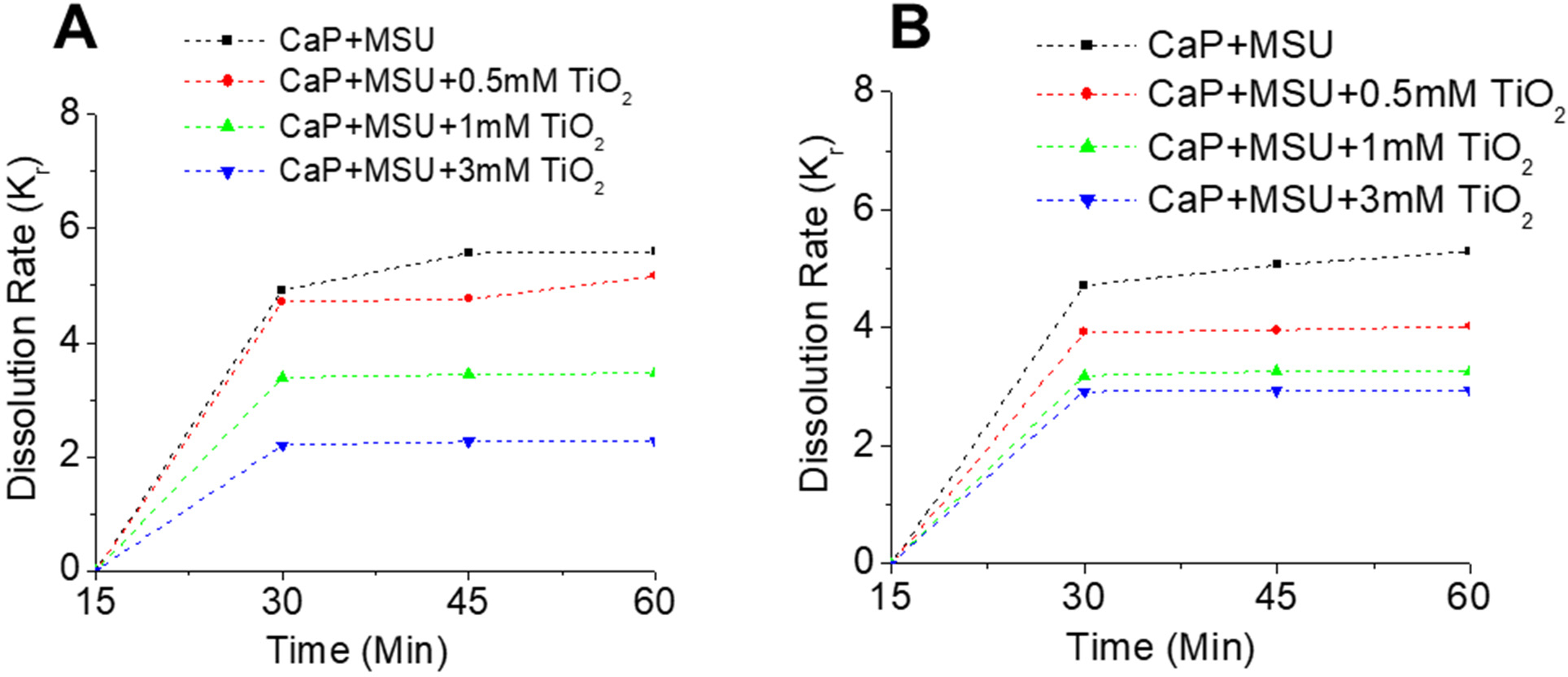
Evaluation of the dissolution rate constant ($K_{r}$) for various crystal groups. $K_{r}$ determination for CaP+MSU and CaP+MSU+TNPs mixed crystals under di-H_2_O conditions (**A**), and in the presence of Hcit (**B**).

The dissolution rate constant was determined for the dissolution of the CaP+MSU and the CaP+MSU+TNPs mixed crystals. The rate constant was calculated based on the constant diffusion-controlled process and specific temperature conditions. The relevance of the kinetic models was for the predictions of possible outcomes for crystal dissolution under prolonged exposure time (t); hence, there is a need to determine the rate constant $\left(K_{r}\right)$. The $K_{r}$ was determined for the dissolution condition in di-H_2_O (Figure 6A) and in Hcit (Figure 6B).

The $K_{r}$ for the crystal dissolution at ΔT = 15 min showed an increase for all the crystal groups, with CaP+MSU+3 mM TNPs having a $K_{r}$ of 2.18, and 3.34 for CaP+MSU+1 mM TNPs, CaP+MSU+0.5 mM TNPs being 4.42, while CaP+MSU had 4.68 (Figure 6A). The low $K_{r}$ value for mixed crystals with high concentrations of TNPs shows the crystal aggregatory property as well as the inhibitory property of high exposures to TNPs for crystal dissolution. At ΔT > 15 min, CaP+MSU+3 mM and CaP+MSU+1 mM TNPs achieved a $K_{r}$ stability of 2.26 and 3.46, respectively. Meanwhile, the mixed crystals with < 0.05 TNPs continued to have an increasing $K_{r}$ (Figure 6A). This further shows that TNPs have stabilization properties on crystal aggregation and inhibit dissolution.

The dissolution $K_{r}$ value for all crystal groups in Kcit was much higher when compared to $K_{r}$ in di-H_2_O (Figure 6A,B). Similar to $K_{r}$ behavior in di-H_2_O, CaP+MSU+3 mM TNPs had a $K_{r}$ value of 2.96, CaP+MSU+1 mM TNPs of 3.10 and CaP+MSU+0.5 mM TNPs of 3.97, while CaP+MSU had 4.79 (Figure 6B). At ΔT > 15min, all mixed crystals maintained a uniform $K_{r}$ value. However, CaP+MSU with no exposure to TNPs continued to increase (Figure 6B). CaP+MSU+3 mM TNPs achieved more stability and a lower $K_{r}$ at ΔT ≥15 in the Hcit solution compared to all crystal groups.

## 4. Discussion

The findings of this study illuminate the significant influence of TNPs on the coaggregation of mixed crystals, particularly CaP and MSU. The images presented in Figure 2A–E, supported by the staining techniques, revealed a notable increase in the aggregation and growth of both CaP and MSU crystals in the presence of TNPs. This phenomenon suggests a robust interplay between these crystal types, with TNPs acting as a catalyst for aggregation. The most pronounced effect was observed in the CaP+MSU+TNPs mixed-crystal samples, as depicted in Figure 1E. These results provide compelling evidence that TNPs plays a pivotal role in facilitating interactions between CaP and MSU crystals, resulting in enhanced aggregation within mixed-crystal formations. A study by Mbanga et al. [32] demonstrated that TNPs could significantly enhance the aggregation of uric acid crystals as they exhibit low dissolution rates and long half-times, indicating their persistent nature in both the body and the environment, potentially posing both short-term and long-term health risks. Their findings support our results, underlining the pro-aggregatory properties of TNPs in the context of crystal-induced diseases. Conversely, a study by Yuan et al. [33] investigated the hydrogen bonding between TNPs and other biological molecules such as proteins, carbohydrates, lipids and other molecules. Their study supports our study on the bonding of CaP and MSU crystals with TNPs leading to crystal aggregation, and hence reveals the complex nature of TNPs’ interactions with different crystal types. This divergence in results underscores the need for a tailored approach when considering TNPs in crystal-related conditions.

The results of these dissolution experiments revealed intriguing dynamics in the behavior of these crystals. Notably, CaP crystals exhibited the highest susceptibility to dissolution, particularly during the initial minute, and this trend continued over the entire 10 min duration (Figure 2A). In contrast, CaP+MSU crystals displayed a significantly higher dissolution rate when compared to the CaP+TNPs group over the entire 10 min period (Figure 2A,D). An interesting finding was the sustained larger size of CaP+MSU+TNPs mixed crystals in comparison to both CaP+MSU and individual crystals until the 10 min mark (Figure 2A–D). This underscores the role of TNPs in stabilizing the mixed-crystal aggregates, ultimately impeding their dissolution.

In comprehensive dissolution experiments involving varying TNPs concentrations (0 mM, 0.5 mM, 1 mM and 3 mM) in the presence of di-H_2_O, the research revealed intriguing findings (Figure 4A–E). The results highlighted a significant difference (*p* < 0.05) in crystal dissolution between CaP+MSU and mixed crystals when 1 mM TNPs was present, particularly after a 10 min exposure (Figure 3A–D). Furthermore, a highly significant difference (*p* < 0.01) was evident at the 10 min mark, emphasizing the concentration-dependent effect of TNPs on crystal dissolution. An interesting observation was that increasing the TiO2 concentration up to 3 mM resulted in a remarkable reduction in the rate of crystal dissolution compared to other mixed crystals (Figure 4A–D), indicative of TNPs’ potential to enhance crystal growth and aggregation, ultimately resulting in reduced dissolution over time. A similar concentration-dependent trend was evident in the presence of HCit, with significant differences in dissolution rates at TNP concentrations exceeding 1 mM, further underscoring the intricate relationship between TNP concentration and crystal dissolution (Figure 4E,G,H). These findings provide valuable insights into the concentration-dependent effects of TNPs on mixed-crystal dissolution, with potential implications for gout disease management.

The dissolution rate constant ($K_{r}$) values observed in our study, particularly at ΔT = 15, highlight intriguing dynamics in the behavior of various crystal groups. In Figure 6A, we noted that CaP+MSU+3 mM TNPs exhibited a $K_{r}$ of 2.18, CaP+MSU+1 mM TNPs showed a $K_{r}$ of 3.34, and CaP+MSU+0.5 mM TNPs displayed a $K_{r}$ of 4.42, while CaP+MSU had a $K_{r}$ of 4.68. These findings underscore the impact of TNPs on crystal dissolution, with higher TNP concentrations contributing to both crystal aggregation and the inhibition of dissolution. At ΔT > 15, the stability of $K_{r}$ values was notably observed in CaP+MSU+3 mM TNPs and CaP+MSU+1 mM TNPs, highlighting the stabilizing properties of TNPs on crystal aggregation (Figure 6A). Additionally, when comparing $K_{r}$ values in HCit to those in diH_2_O (Figure 6B), we observed that $K_{r}$ values were consistently higher in HCit. CaP+MSU+3 mM TNPs demonstrated a $K_{r}$ of 2.96, CaP+MSU+1 mM TNPs exhibited a $K_{r}$ of 3.10, CaP+MSU+0.5 mM TNPs showed a $K_{r}$ of 3.97, and CaP+MSU had a $K_{r}$ of 4.79. These findings imply that HCit had a pronounced effect on $K_{r}$, and the introduction of TNPs led to enhanced stability and reduced $K_{r}$ values, particularly in CaP+MSU+3 mM TNPs, indicating TNPs’ potential to stabilize mixed-crystal aggregates, especially under the influence of HCit.

Our results were mainly focused on the influence of TNPs on the aggregation or dissolution of CaP and MSU crystals, synthesized in our laboratory. In support of our study, Karalkeviciene et al. have shown that the addition of organic compounds, such as dodecanedioic acid, DL-aspartic acid and suberic acid, could contribute to the formation of calcium-deficient hydroxyapatite, depending on the organic additive type and concentration [34]. Although the specific shape of the CaP crystals and its influence on the growth were not mentioned in this study; the pure CaP crystals that were formed were in brushite or amorphous configurations, prior to the addition of other crystals [28,30]. The mechanism of co-crystallization includes the nucleation of stone constituent crystals, their growth or aggregation to a size that can interact with some intrarenal structure, their retention within the system and their further aggregation and/or secondary nucleation to form a clinical knee-joint crystal [27]. Our goal was to look at the role of TiO_2_ as an environmental contaminant in the crystallization of MSU+CaP, which may be secondary to the initial concentrations of individual ions [28].

In summary, our interest in this research is primarily rooted in the potential implications for MSU crystal-induced diseases, particularly gout, and we propose that TNP’s stabilization could be enhanced by CaP-mixed MSU crystals. The in vitro approach was chosen as an initial step to circumvent the complexities associated with biological variables, including cellular components. This approach allowed us to focus solely on the interactions between TNPs and mixed crystals. Based on the promising results of this study, our future direction will involve transitioning to a biological environment to explore how these interactions may impact the progression of gout or other crystal-related conditions.

From a healthcare perspective, this study holds significant implications. Gout, characterized by crystal deposition and aggregation, is a painful and debilitating condition. Understanding how TNPs impact crystal behavior could lead to potential therapeutic interventions that target crystal stability. By inhibiting crystal aggregation and enhancing dissolution, TNP-related pathogenesis may offer new avenues for treating or preventing gout and similar crystal-induced diseases. Although, cellular effects on TNPs were found to be heterogenic and produced in vivo consequences, challenging healthy bone tissue in well-fixed orthopedic implants, and can contribute to pathological mechanisms underlying the aseptic loosening of titanium-based metal implants [35]. Thus, our results present the possibility of TNPs’ influence on CaPs’ interaction with MSU in those conditions. Looking forward, expanding this research into more biologically active microenvironments to assess the true impact of TNPs on crystal-induced pathophysiology and the impact on the behavior of the co-crystals’ incorporation of porosity into the model will be the next step.

Finally, our study illuminates the intricate dynamics of TNPs in the context of mixed-crystal aggregation and dissolution. While our in vitro approach provides valuable insights, it also highlights the need for further research in biological settings. The potential implications for healthcare are promising, and we look forward to unraveling the full extent of TNPs’ impact on crystal-induced diseases, opening new doors for therapeutic advancements.

## 5. Conclusions

This study delved into the complex interactions between TNPs and mixed crystals comprising CaP and MSU. The research was motivated by the relevance of crystal aggregation and dissolution in crystal-induced diseases, with a particular focus on gout. The findings showcased that TNPs significantly increased the formation of mixed CaP+MSU crystals and played a pivotal role in stabilizing them. The dissolution experiments revealed that TNPs effectively inhibited the dissolution of mixed crystals, especially in the presence of a crystal inhibitor, HCit. Understanding how TNPs can stabilize mixed crystals and hinder their dissolution offers new possibilities for therapeutic interventions. These results open doors to novel approaches in the treatment and prevention of gout and other crystal-related conditions.

## Data Availability

The data presented in this study are available in the article.
